# Electroacupuncture ameliorates postoperative cognitive dysfunction and associated neuroinflammation via NLRP3 signal inhibition in aged mice

**DOI:** 10.1111/cns.13784

**Published:** 2021-12-23

**Authors:** Long Sun, Yue Yong, Pan Wei, Yongqiang Wang, He Li, Yalan Zhou, Wenqing Ruan, Xing Li, Jiangang Song

**Affiliations:** ^1^ Department of Anesthesiology Shuguang Hospital Affiliated with Shanghai University of Traditional Chinese Medicine Shanghai P.R. China; ^2^ Research Institute of Acupuncture Anesthesia Shuguang Hospital Affiliated with Shanghai University of Traditional Chinese Medicine Shanghai P.R. China

**Keywords:** neuroinflammation, NF‐κB, NLRP3 inflammasome, postoperative cognitive dysfunction

## Abstract

**Background:**

Postoperative cognitive dysfunction (POCD) is associated with worsened prognosis especially in aged population. Clinical and animal studies suggested that electroacupuncture (EA) could improve POCD. However, the underlying mechanisms especially EA’s regulatory role of inflammasomes remain unclear.

**Methods:**

The model of POCD was established by partial hepatectomy surgery in 18‐month mice with or without postoperative EA treatment to the Baihui acupoint (GV20) for 7 days. Cognitive functions were assessed by Morris water maze test, and proinflammatory cytokines IL‐1β and IL‐6 and microglia activity were assayed by qPCR, ELISA, or immunohistochemistry. Tight junction proteins, NLRP3 inflammasome and downstream proteins, and NF‐κB pathway proteins were evaluated by western blotting.

**Results:**

EA markedly preserved cognitive dysfunctions in POCD mice, associated with the inhibition of neuroinflammation as evidenced by reduced microglial activation and decreased IL‐1β and IL‐6 levels in brain tissue. EA also preserved hippocampal neurons and tight junction proteins ZO‐1 and claudin 5. Mechanistically, the activation of NLRP3 inflammasome and NF‐κB was inhibited by EA, while NLRP3 activation abolished EA’s treatment effects on cognitive function.

**Conclusion:**

EA alleviates POCD‐mediated cognitive dysfunction associated with ameliorated neuroinflammation. Mechanistically, EA’s treatment effects are dependent on NLRP3 inhibition.

## Graphical abstract

EA alleviates PND‐mediated cognitive dysfunction associated with ameliorated neuroinflammation. Mechanistically, EA’s treatment effects are dependent on NLRP3 activation.

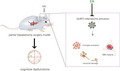

## 1 INTRODUCTION

Postoperative cognitive dysfunction (POCD) is primarily characterized by postoperative memory impairment, reduced information processing ability, and executive dysfunction. POCD is a common and serious complication induced by major surgery, especially in elderly people. The reported incidence ranges from 15% to 60%.^1^, ^2^ In addition, the cognitive dysfunction following POCD may not be transient and can persist for several years, potentially increasing the incidence of Alzheimer's disease (AD).^3^ However, the pathophysiology of POCD remains unknown. A large number of studies have shown that POCD involves neuronal apoptosis, neurogenesis decline, impairment of synaptic plasticity, and neurodegeneration induced by neuroinflammation and oxidative stress in the central nervous system (CNS).^3^, ^4^

A growing body of evidence indicates that neuroinflammation plays an important role in the onset and progression of POCD. Lots of inflammatory molecules have been identified in both animal models and POCD patients. Inflammation associated with POCD involves both central and peripheral compartments.^5^, ^6^ Surgery activates an immune response and results in the release of systemic proinflammatory factors and eventually leads to neuroinflammation.^7^, ^8^, ^9^ Studies have shown that microglia are activated by peripheral proinflammatory cytokines^10^ and can release additional proinflammatory cytokines, exacerbating neuroinflammation and brain damage.^11^ Once activated by proinflammatory cytokines in the brain, microglia tend to separate into two “polarizing” phenotypes M1 and M2, sometimes associated with the release of cytokines. The proinflammatory functions of M1‐type macrophages are associated with the secretion of the proinflammatory cytokines IL‐1, TNF‐α, and IL‐6, while M2 macrophages are broadly seen as antiinflammatory type of macrophage.^12^ The hippocampus, a memory and learning center, extensively expresses the IL‐6 and IL‐1β receptors.^13^ This is a double‐edged sword, such that the proinflammatory factor receptors play a key role in normal studying and memory. However, under pathological conditions, the hippocampus is particularly vulnerable to proinflammatory factors. Therefore, the release of proinflammatory cytokines by M1‐type microglia may lead to the injury of hippocampus and eventually lead to POCD.^7^

Nucleotide‐binding leucine‐rich repeat‐containing receptors (NLR), or receptors that respond to damage‐associated molecular patterns (DAMPs), produced during tissue injury.^14^, ^15^ The most widely studied NLRs are inflammasome‐forming NLRs, among which inflammasome complexes (NLRP1, NLRP3, NLRC4, and AIM2) have been well identified in the process of inflammation.^16^, ^17^ Upon activating DAMPs, these NLRs promote the release of proinflammatory cytokines such as IL‐1β and IL‐18. Lots of studies have suggested that anesthesia induces IL‐1β and IL‐18 secretion and promotes the neuroinflammation and neurotoxicity via the NLRP3 inflammasome activation in the hippocampus of the animal model.^18^, ^19^ Besides, studies have shown that antiinflammatory drugs and IL‐1β receptor antagonists can decrease the inflammatory factors’ expression in the hippocampus and alleviate the cognitive dysfunction induced by surgery.^20^, ^21^ However, lots of antiinflammatory drugs were reported significant adverse effects.^22^, ^23^ Thus, it is particularly important to explore new therapeutic strategies with fewer adverse effects in the POCD clinical treatment.

Acupuncture is one of the most popular traditional Chinese therapeutic techniques, where thin needles are inserted into different acupoints to treat lots of diseases.^24^, ^25^ Electroacupuncture (EA) is an alternative acupuncture technique. It applies an electric current to the inserted needles.^26^ EA stimulation was proved to prevent cognitive dysfunction in a mouse model of Alzheimer's disease (AD) by inhibiting synaptic degeneration and neuroinflammation.^27^ In addition, EA treatment can improve the learning and memory function in LPS‐treated mice by enhancing the expression of α7‐nAChR and cholinergic factors and suppressing neuroinflammation in the hippocampus.^28^ Previous clinical studies demonstrated that EA treatment reduced the incidence of POCD and the levels of systematic inflammatory markers in aged patients.^29^, ^30^ In vivo animal studies suggested that EA improved cognitive impairment.^31^, ^32^ However, the role of NLRP3 inflammasome in therapeutic EA of POCD is still unclear. In the current study, we tried to demonstrate the effects of EA on POCD and verified whether NLRP3 inflammasome is involved in the improvement of cognitive function in aged mice by EA treatment.

## 2 METHODS

### 2.1 Animals and grouping

Male C57BL/6 mice (18 months old, 24–35 g weight) were purchased from the Leagene Biological (Shanghai, China). The mice were housed in a humidity‑ and temperature‑controlled room at a 12:12 h light/dark cycle with free access to laboratory food and water. The experimental procedures were carried out according to the “Guide for the Care and Use of Laboratory Animals” published by the NIH, and the study was approved by the Animal Care and Use Committee of the Shanghai University of Traditional Chinese Medicine.

Mice were randomly assigned into sham (no surgery or EA), POCD (partial hepatectomy), and POCD + EA groups. The 70% partial hepatectomy surgeries were carried out according to previously described.^33^ Briefly, under isoflurane (Sigma) anesthesia, the median and left lobes, 70% of the total liver, were tied at the origins of the lobes and then surgically removed. The peritoneum and skin were reapproximated with a running suture.

### 2.2 Intracerebroventricular injection

The NLRP3 activator nigericin or inhibitor (MedChemExpress) was given by intracerebroventricular injection as previously described.^34^ Briefly, 4 µg nigericin or 2 µg MCC950 was dissolved in 2 µl vehicle (normal saline: DMSO = 9:1) 2 µl drug solution or vehicle was infused at a rate of 0.2 µl/min by 10‐µl Hamilton syringe 1 h after POCD modeling. The stereo coordinates for lateral ventricle injection were lateral to the bregma 1.0, 3.0 mm under the horizontal plane of the bregma.

### 2.3 Electroacupuncture (EA)

In POCD + EA group, the Baihui acupoint (GV20) was selected, and EA was performed based on Guo et al. with minor modifications.^35^ In brief, the day after the POCD surgery, mice were immobilized and the stainless‐steel needle (0.16 mm × 7 mm) was inserted into the GV20 vertically with a depth of 2 mm. The end of the needle was attached to the electrode from an EA device, HANS acupoint nerve stimulator (HANS‐LH202H). The parameters of EA were set as follows: intensity 0.5 mA and frequency: 2 Hz. The EA stimulation was performed for 20 min each time and twice daily for 7 days. The control and POCD groups were immobilized but not EA stimulated.

### 2.4 Morris water maze (MWM)

The cognitive and memory function of mice was evaluated by MWM experiment as previously described.^36^ In brief, the mice were subjected to training tests for five consecutive days the week before surgery. During the training process, the mice were guided to reach the hidden platform. Then, the space exploration test was performed one‐week post POCD surgery. Briefly, the platform was removed, and then, the mice were put into water to swim without any interruption for 60 s. The swimming paths were recorded.

### 2.5 Reverse transcription‐PCR (qPCR)

Total RNA from mouse hippocampus tissues was extracted with Trizol reagent (Sigma‐Aldrich). Reverse transcriptional PCR was performed using the iScripe™ cDNA Synthesis kit (Bio‐Rad). qPCR analysis was performed using the QIAGEN One‐Step qPCR kit (QIAGEN) on the ABI 7500 PCR System. The conditions for the qPCR analysis were 95°C for 10 min, followed by 37 cycles of 95°C for 10 s and 60°C for 60 s. The fold changes of RNA transcripts were calculated by the 2^−ΔΔCt^ method, and the 18s was used as an internal gene. All the primer sequences are listed in Table 1.

**TABLE 1:** Sequences of rat‐specific primers uses in RT‐PCR for IL‐1β, IL‐6, NLRP1, NLRP3, AIM2, and NLRP4

Genes	Primers
IL‐1β	F: 5′‐GCAsACTGTTCCTGAACTCAACT‐3′
	R: 5′‐ATCTTTTGGGGTCCGTCAACT‐3′
IL‐6	F: 5′‐TCCAGTTGCCTTCTTGGGACTGA‐3′
	R: 5′‐TAAGCCTCCGACTTGTGAAGTGGT‐3′
NLRP1	F: 5′‐GCCCTGGAGACAAAGAATCC‐3′
	R: 5′‐AGTGGGCATCGTCATGTGT‐3′
NLRP3	F: 5′‐ATTACCCGCCCGAGAAAGG‐3′
	R: 5′‐TCGCAGCAAAGATCCACACAG‐3′
AIM2	F: 5′‐TGGCAAAACGTCTTCAGGAGG‐3′
	R: 5′‐AGCTTGACTTAGTGGCTTTGG‐3′
NLRP4	F: 5′‐AGACTCGTCACGAAGGGAGA‐3′
	R: 5′‐ATAAAACCTCATCCCTGTCTATGT‐3′

### 2.6 Enzyme‐linked immunosorbent assay (ELISA)

The protein levels of IL‐1β (Abcam) and IL‐6 (Abcam ) in the hippocampus tissue extracts were measured according to the manufacturer's protocol. In brief, the hippocampus tissue was dissected and homogenized using homogenizer in ice‐cold lysis buffer containing HEPES 25 mM, pH 7.4, 3‐[(3‐cholamidopropyl) dimethyl‐ammonio]‐1‐propanesulfonate 0.1%, MgCl_2_ 5 mM, EDTA 1.3 mM, EGTA 1 mM, 10 μg/ml pepstatin, aprotinin, and leupeptin, and 1 mM PMSF. The homogenates were centrifuged at 10,000 *g* for 15 min, and supernatants were used for ELISA assay.

### 2.7 Western blotting

The hippocampal tissues were lysed in RIPA buffer (Millipore), and total protein concentrations were quantified by BCA Protein Assay Kit (Thermo Fisher Scientific) according to the manufacturer's instructions. A total of 20 µg proteins were loaded to an 8% SDS‐PAGE and then transferred onto PVDF membranes (Millipore). Then, the membranes were blocked with 5% nonfat milk at room temperature for 2 h and then incubated with p65 (1:2000, ab32536, Abcam), p‐p65 (1:2000, ab194726, Abcam), p‐IκB‐α (1:1000, ab92700, Abcam), IκB‐α (1:2000, ab32518, Abcam), NLRP3 (1:1000, ab214185, Abcam), ASC (Santa Cruz Biotechnology), Caspase‐1 (1 µg/ml, ab138483, Abcam), iNOS (1:1000, ab178945, Abcam), CD206 (1:1000, PA5‐46994, Thermo Fisher) overnight at 4°C. Secondary antibodies are HRP‐conjugated against rabbit or mouse (Abcam). Membranes were then incubated with chemiluminescent substrates (ThermoFisher), and images were captured in iBright CL750 (ThermoFisher) and ImageJ software and were used for gel analyses.

### 2.8 Immunohistochemistry (IHC)

The hippocampal tissues were fixed in 4% paraformaldehyde, embedded in paraffin, and cut into 4‐µm‐thick sections. The slices were deparaffined and rehydrated with graded series of ethanol. Then, the slices were immersed in 3% H_2_O_2_, incubated at room temperature for 10 min, and washed with PBS for three times. The slices were incubated with primary antibodies anti‐NLRP3 (1:200, ab214185, Abcam), Iba1 (1:2000, ab178846, Abcam), NeuN (1:1000, ab177487, Abcam), at 4℃ overnight, and following incubated with secondary antibody (Goat Anti‐Rabbit IgG 1:500, Abcam). Then, the slices were coated with 100 μl DAB buffer (Thermo Fisher Scientific) and stained with hematoxylin for 1–2 min.

### 2.9 Statistical analysis

All the data are shown as the mean ± standard deviation (SD). Statistical analysis was carried out using SPSS 21.0 software (IBM Corp.). The significance between two groups was analyzed using one‐way ANOVA followed by Tukey‐Kramer multiple comparisons test or Student's unpaired *t* test. Before statistical analysis, the data were tested for Gaussian distribution assessed using Shapiro–Wilk normality test after transform to logarithms. *p* < 0.05 was considered to indicate a statistically significant difference.

## 3 RESULTS

### 3.1 EA treatment improved the cognitive impairment of aged POCD mice

**FIGURE 1:**
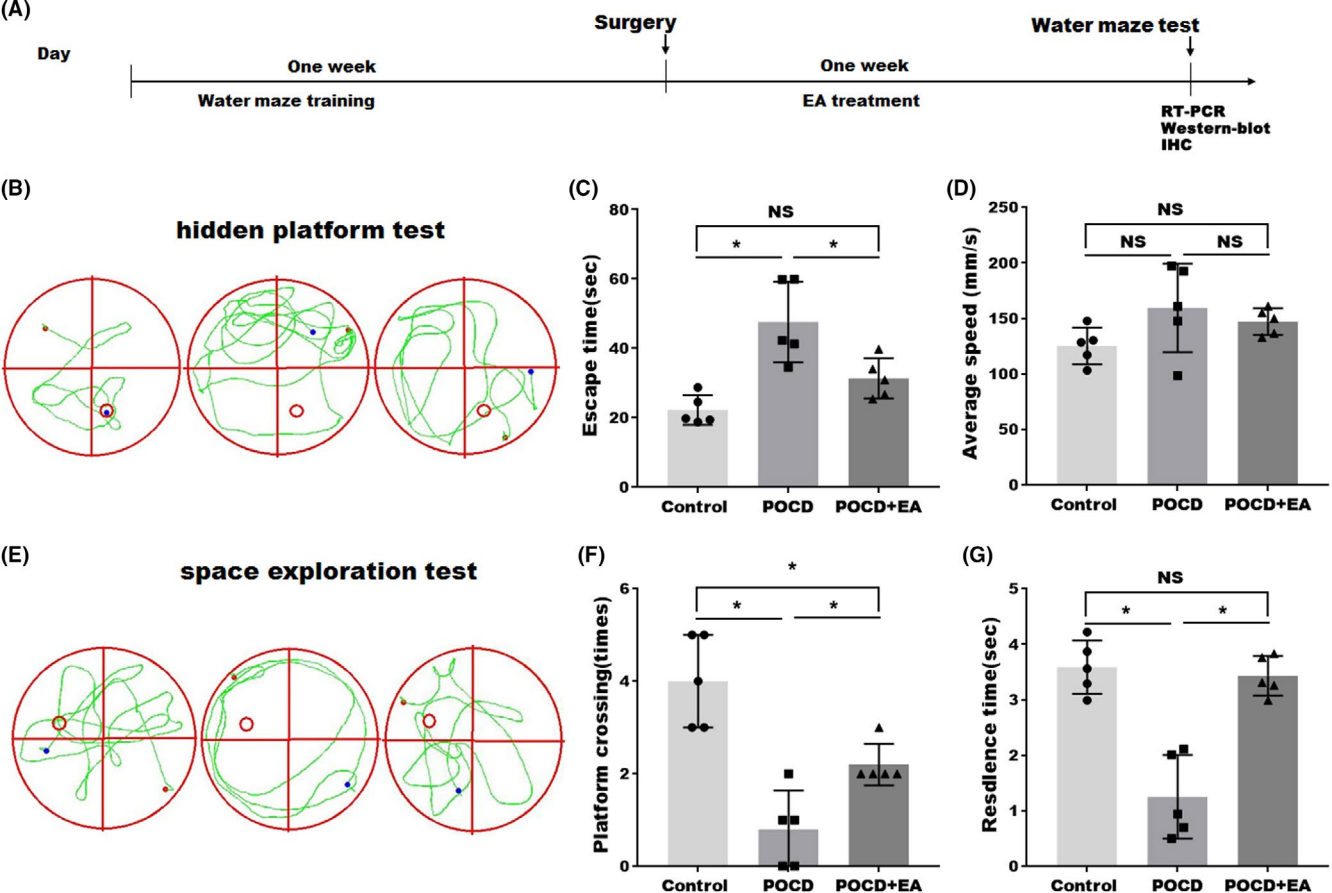
EA treatment improved the cognitive functions of aged POCD mice. (A) Diagram showing the timeline for Morris water maze (MWM) training, postoperative tests, and EA treatment. (B, E) Effects of EA treatment on postoperative cognition were analyzed by MWM. (C, D) Hidden platform training test assessing the memory and (F, G) space exploration experiment assessing the learning. Each data point was mean ± SD (*n* = 5). *p < 0.05

To test whether EA treatment could improve the cognitive functions in POCD mice, MWM test was performed (Figure 1A), including a hidden platform test for memory (Figure 1B) and space exploration test for learning (Figure 1E). In the hidden platform test, POCD mice exhibited longer escape latency compared with the control group, which was significantly shortened by EA treatment (Figure 1C), suggesting that EA improved memory in aged POCD mice. There was no significant difference in swimming speed between sham and POCD groups, indicating that POCD did not affect the motor function of mice (Figure 1D).

In the space exploration experiment (Figure 1D), the residence time of the animal stayed in the target quadrant and number of the platform crossing time were significantly reduced in POCD mice, while the effect was restored by EA treatment (Figure 1F,G), suggesting that EA treatment improved learning capabilities in aged POCD mice. Taken together, it was suggested that EA treatment can alleviate neurological dysfunction in aged POCD mice.

### 3.2 EA treatment decreased hippocampal neuroinflammation and preserved hippocampal neurons of aged POCD mice

**FIGURE 2:**
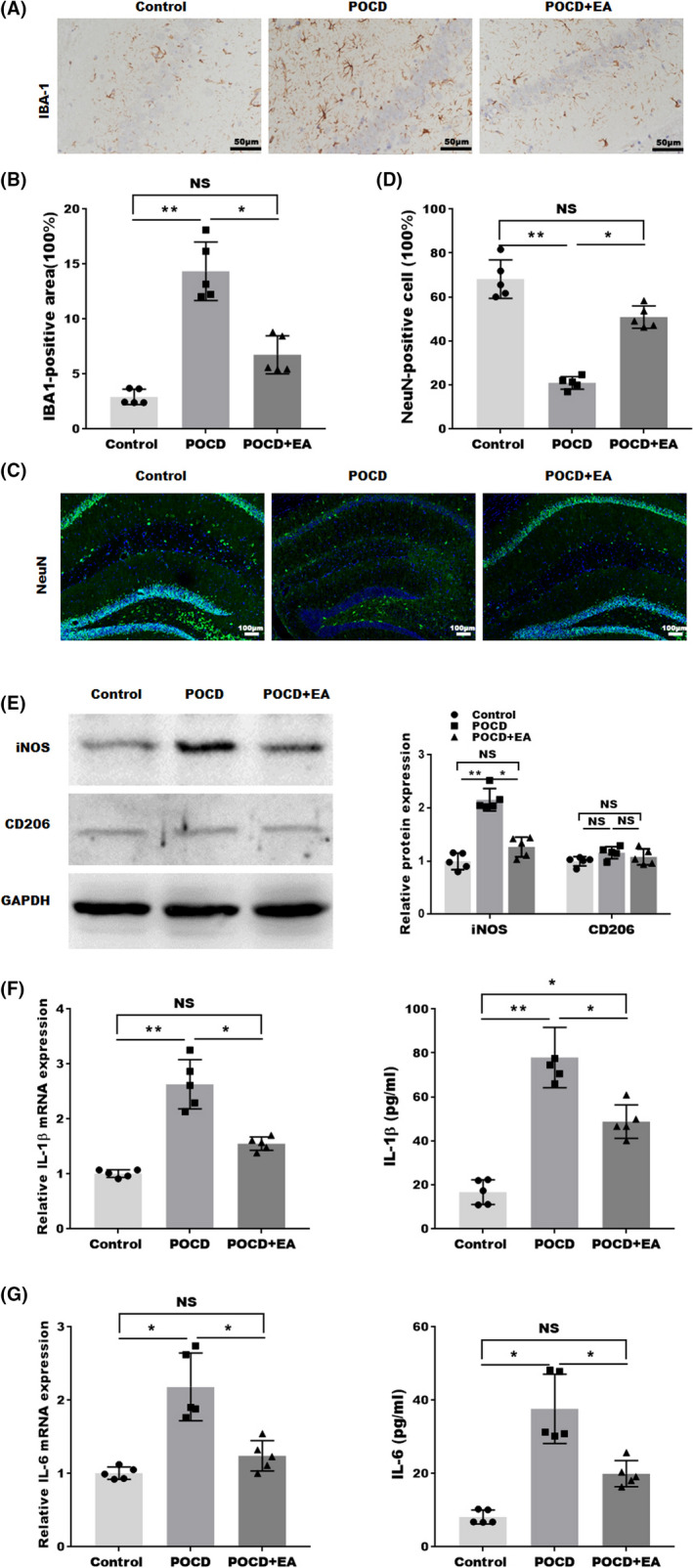
EA treatment decreased neuroinflammation in the hippocampus of aged POCD mice. (A) Representative images of Iba1 staining and (B) quantification of Iba1‐positive area in hippocampus. (C) Representative images (NeuN: green; DAPI: blue) and (D) quantifications of nucleus staining in the hippocampus. (E) Western blotting and semiquantifications for iNOS and CD206 protein level in hippocampal tissues. (F) The mRNA and protein expression IL‐1β in the hippocampal tissue detected by qPCR and ELISA from hippocampal tissues. (G) The mRNA and protein expression IL‐6 in the hippocampal tissue were detected by qPCR and ELISA from hippocampal tissues. Each data point was mean ± SD (*n* = 5). *p < 0.05, **p < 0.01

Neuroinflammation associated with surgical trauma is a crucial pathogenic factor in POCD. As the brain‐resident myeloid cells, microglia play a key role in mediating neuroinflammation. To this end, we labeled microglia with Iba1 at 1‐week postsurgery. As shown in Figure 2A,B, more microglia cells were labeled in the hippocampus of the aged POCD mice, while EA treatment significantly decreased the microglia labeling after POCD. These data suggest the presence of microglial accumulation in aged POCD hippocampus, which is inhibited by EA treatment. To test whether the inhibition of neuroinflammation is associated with neuronal preservation, we assessed hippocampal neuronal numbers. As shown in Figure 2C,D, immunofluorescence suggested that POCD decreased the number of NeuN‐positive neurons in the hippocampus, which was restored by EA treatment, suggesting that EA treatment suppressed the neuronal loss induced by POCD. Previous studies suggested that the polarization of microglia plays an important role in the pathophysiological process of neuroinflammation.^37^ Therefore, the expression of M1 polarization marker iNOS and M2 polarization marker CD206 in the hippocampi was analyzed by western blotting. As expected, Figure 2E shows that the M1‐type microglia were significantly increased in POCD group, while the effect was blocked by EA treatment. Unlike the results of M1 polarization, there are no difference in M2 polarization among control, POCD, and POCD with EA treatment group.

Neuroinflammation is also characterized by increased proinflammatory cytokines in the brain. Thus, the levels of inflammatory factors such as IL‐1β and IL‐6 in the hippocampi were analyzed by qPCR and ELISA 1‐week postsurgery. As shown in Figure 2F,G, the mRNA and protein levels of IL‐1β and IL‐6 were markedly increased in hippocampal tissues in aged POCD mice, which were attenuated by EA treatment. These data suggested that EA treatment can alleviate neuroinflammation in aged POCD mice.

### 3.3 EA treatment suppressed the activation of NLRP3 inflammasome associated with the inhibition of NF‐κB pathway

**FIGURE 3:**
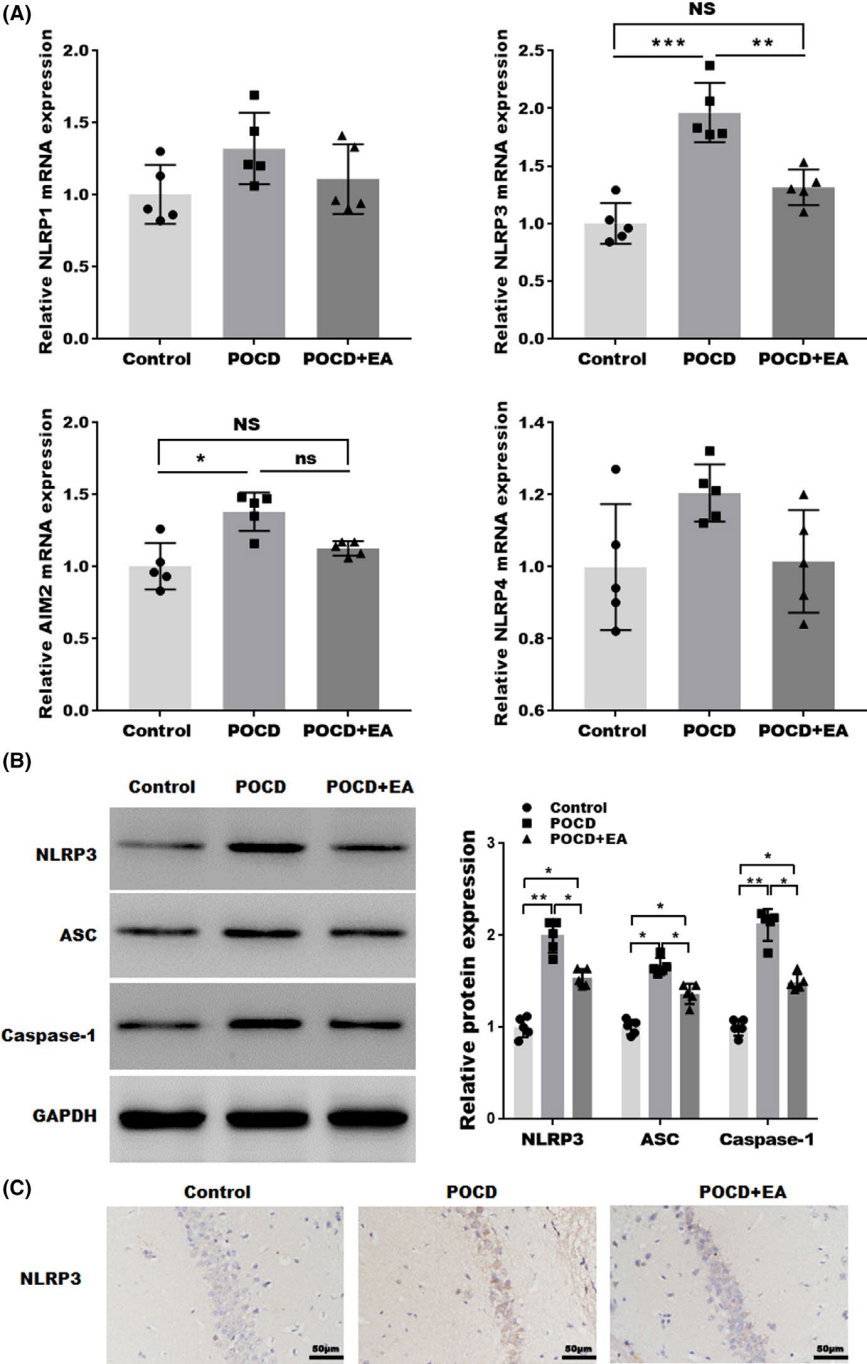
EA treatment suppressed the activation of NLRP3 inflammasome. (A) qPCR analysis of NLRP1, NLRC4, NLRP3, and AIM2 inflammasome mRNA levels in the hippocampal tissues. (B) Western blotting and semiquantifications for NLRP3, ASC, and Caspase‐1 protein levels in hippocampal tissues. (C) IHC staining for NLRP3 in hippocampal tissues. Each data point was mean ± SD (*n* = 5). *p < 0.05, **p < 0.01

Since the inflammasome signaling pathway is a major mediator neuroinflammatory process in various CNS diseases,^38^, ^39^ we evaluated the activation of NLRP1, NLRP3, NLRC4, and AIM2 inflammasome in aged POCD mice. As shown in Figure 3A, we observed significant increase in NLRP3 and AIM2 mRNA levels, but not in NLRP1 or NLRP2 mRNA levels. Besides, only the increased NLRP3 expression could be inhibited by EA treatment. This is further confirmed at the protein level by western blotting (Figure 3B) and IHC (Figure 3C). In addition, as shown in Figure 3B, the recruitment of the adaptor protein apoptosis‐associated speck‐like protein containing a CARD (ASC) and caspase‐1 by NLRP3 inflammasome was also increased in aged PDN hippocampus, which was partially reversed by EA treatment (Figure 3B). These data suggested that EA treatment can inhibit the activation of NLRP3 inflammasome in aged POCD mice.

**FIGURE 4:**
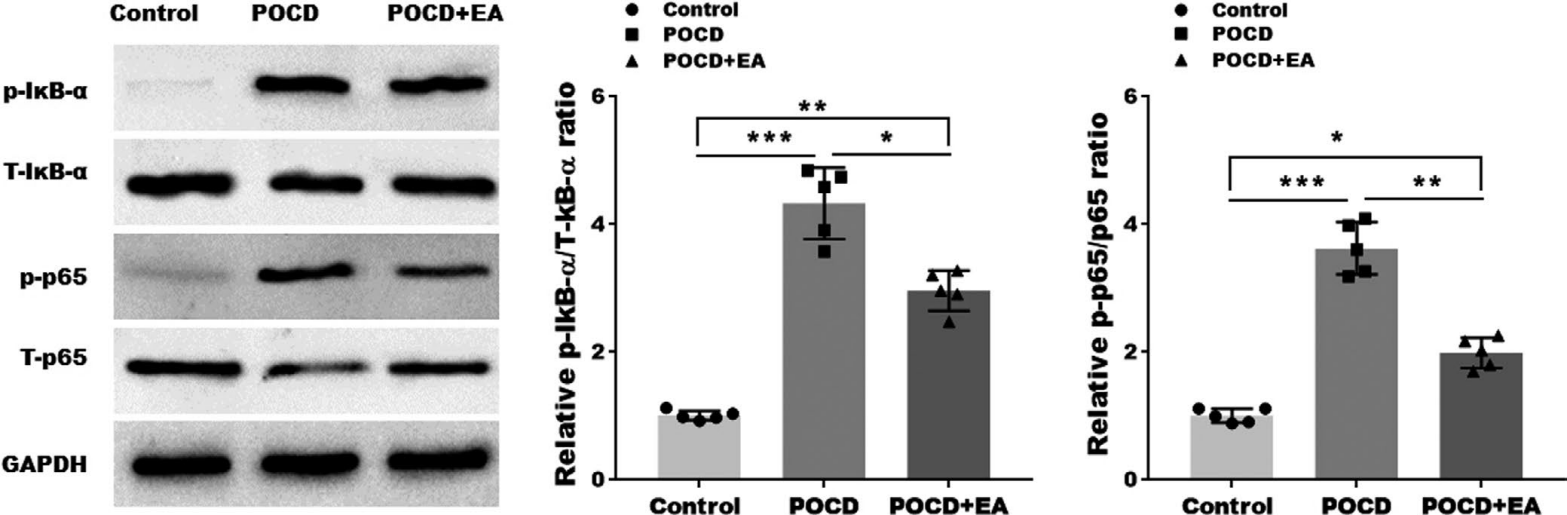
EA treatment inhibits the NF‐κB pathway. Western blotting and semiquantification showing the protein expressions of phospho‐IκB subunit α (p‐IκB‐α), total IκB‐α, phosphor‐p65 (p‐p65), and total p65 in the hippocampal tissues. Each data point was mean ± SD (*n* = 5). *p < 0.05, **p < 0.01

Emerging evidence suggests the critical role of NF‐κB pathway in neuroinflammation and NLRP3 activation. To evaluate the effect of EA treatment on NF‐κB signaling, we analyzed the protein levels of NF‐κB pathway molecules. As shown in Figure 4, the expression levels of phosphorylated IκB‐α and p65 were markedly increased in POCD mice, which was partially reversed by EA treatment. These results indicated that, along with suppressed NLRP3 activation, EA treatment also inhibited NF‐κB signaling, which may serve as the mechanism for NLRP3 inhibition.

### 3.4 NLRP3 inflammasome is critical for the treatment effects of EA

**FIGURE 5:**
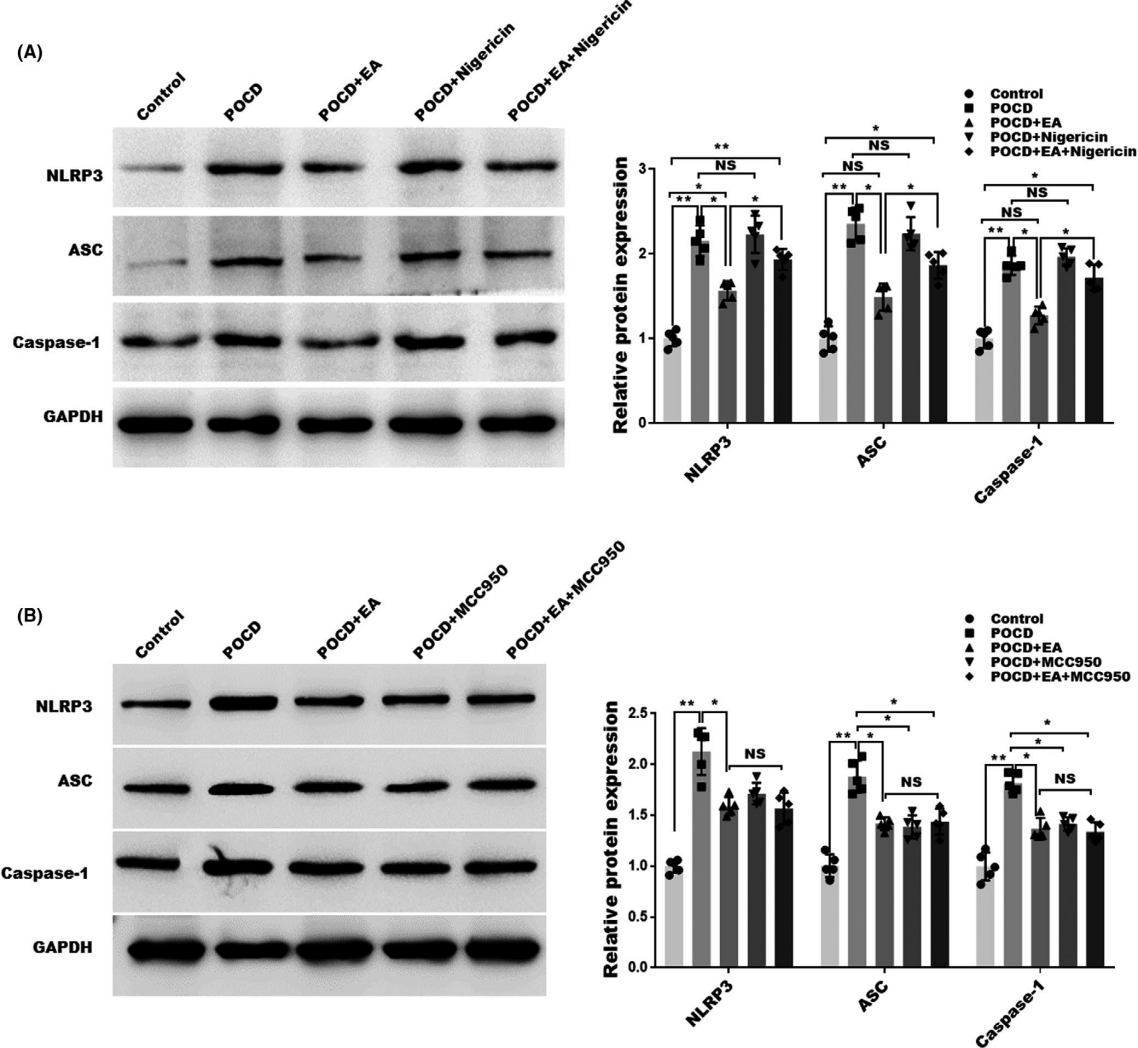
Effects of nigericin on NLRP3 activation. (A)Western blotting and semiquantification for NLRP3, ASC, and Caspase‐1 expression in hippocampus tissues of POCD mice treated with or without NLRP3 activator nigericin. (B) Western blotting and semiquantification for NLRP3, ASC, and Caspase‐1 expression in hippocampus tissues of POCD mice treated with or without NLRP3 inhibitor MCC950. Each data point was mean ± SD (*n* = 5). *p < 0.05, **p < 0.01

We then asked whether the inhibition of NLRP3 inflammasome is critical for EA’s treatment effects. To this end, NLRP3 inflammasome was forcefully activated by nigericin, an activator of NLRP3. As shown in Figure 5A, as detected by western blotting, nigericin cannot further increase NLRP3, ASC, or Caspase‐1 in POCD mice. Nevertheless, nigericin administration partially abolished the EA’s inhibition on NLRP3, ASC, and Caspase‐1. To further confirm the NLRP3 is critical for the protective effect of EA on the development of POCD, POCD mice were treated with EA in the presence or the absence of MCC950, an inhibitor of NLRP3. As shown in Figure 5B, as detected by western blotting, NLRP3, ASC, and caspase‐1 were significantly increased in the POCD mice model, while this phenomenon was reversed by EA in the presence or the absence of MCC950. These data validate the activation and the inhibition of NLRP3 by nigericin and MCC950 in our system.

**FIGURE 6:**
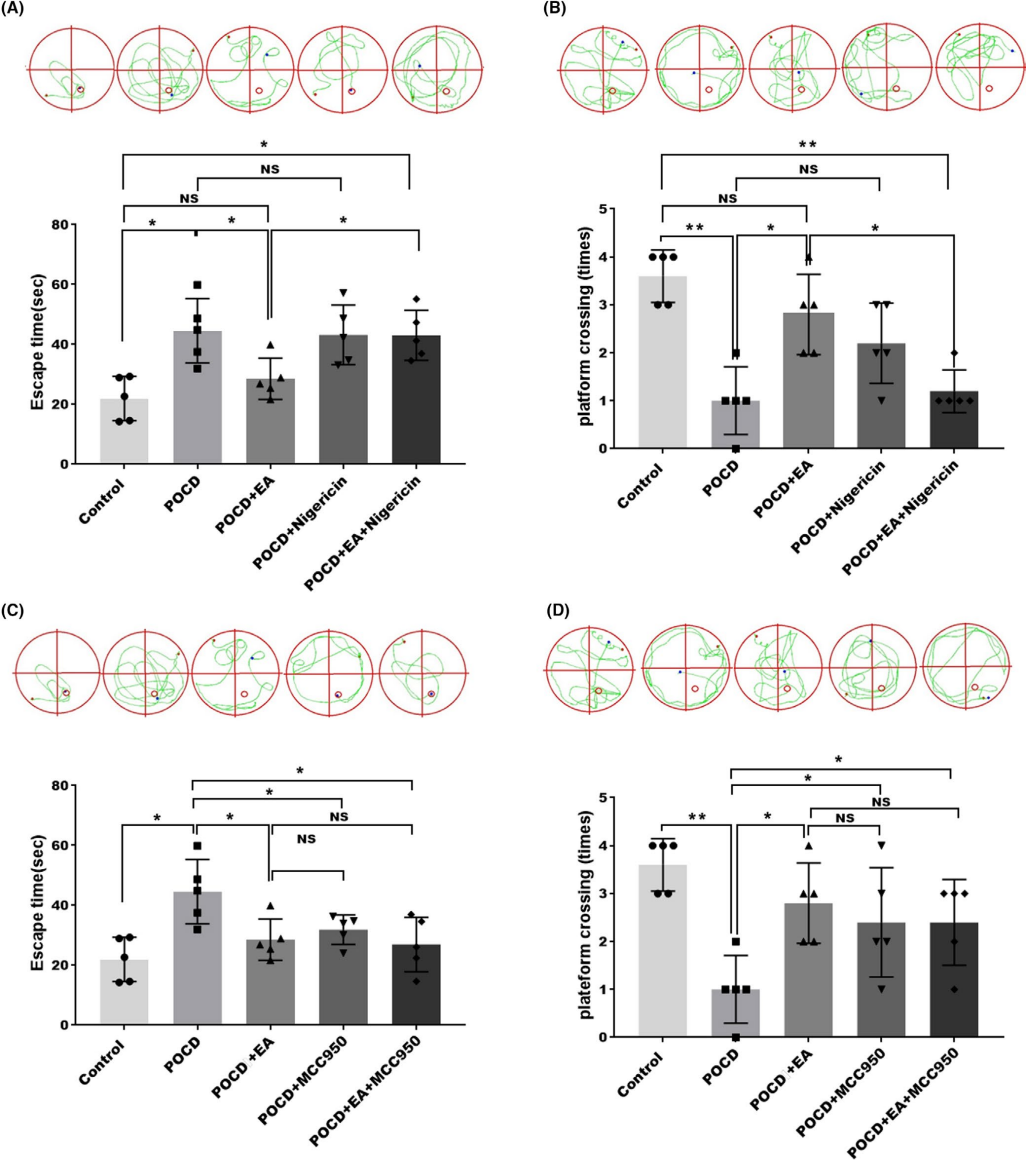
Effects of nigericin on cognitive function in POCD mice. (A, B) Effects of NLRP3 activator nigericin and EA treatment on postoperative cognition was analyzed by MWM in POCD mice model. (C, D) Effects of NLRP3 inhibitor MCC950 and EA treatment on postoperative cognition were analyzed by MWM in POCD mice model. Each data point was mean ± SD (*n* = 5). (A, C) Hidden platform training test. (B, D) Space exploration experiment. *p < 0.05, **p < 0.01 (*n* = 5)

We next explore the effects of nigericin and MCC950 on cognitive function using the WMW test. As expected, nigericin did not further worsen the cognitive functions as indicated by escape latency (Figure 6A) and platform crossing (Figure 6B), but markedly abolished the treatment effects afforded by EA (Figure 6A,B). Strikingly, we found that MCC950 and EA treatment exhibited similar effects on alleviating neurological dysfunction in POCD mice as indicated by escape latency (Figure 6C) and platform crossing (Figure 6D), However, EA combined with MCC950 treatment did not further increase platform crossing time in PND mice compared with MCC950 alone. These data suggesting that EA treatment effects are dependent on EA’s inhibition on NLRP3 inflammasome.

## 4 DISCUSSION

POCD is characterized as a persistent or transient impairment of cognition, including information processing, consciousness, memory, and social ability, in patients undergoing anesthesia surgery.^40^ Recently, studies suggested that neuroinflammation plays a critical role in the development of POCD, and EA stimulation can improve the cognitive function of POCD.^31^ However, the underlying mechanisms of EA treatment on the cognitive improvement are still unclear. In the current study, we provided evidence that EA ameliorates neuroinflammation‐associated cognitive impairment and an aged POCD model. The following findings were proved by our results. (1) EA treatment improved cognitive impairment of POCD in aged mice; (2) EA treatment decreased neuroinflammation in mouse hippocampus; (3) EA treatment suppressed the activation of NLRP3 inflammasome; and (4) NLRP3 inflammasome is critical for the treatment effects of EA.

Aging per se is a risk factor for the development of POCD, which is associated with potential long‐term sequela.^41^ It is of more significant importance to evaluate POCD and its underlying mechanisms in aged individuals. In the present study, we assessed POCD in aged (18‐month‐old) mice, in order to unveil novel mechanisms and potential treatment targets for this high‐risk population.

Neuroinflammation plays an important role in POCD pathogenesis.^42^ Surgery leads to marked increase in the proinflammatory cytokines such as IL‐1β, TNF‐α, and IL‐6 in the hippocampal tissues. These proinflammatory cytokines can further lead to the M1 polarization of microglial cells.^43^, ^44^ The M1‐type macrophage further releases the proinflammatory cytokines such as IL‐1β, TNF‐α, and IL‐6, thereby resulting in brain damage and cognitive impairment.^45^ It has been found that knockdown of IL‐1R1‐expressing CX3CR1^+^ cells in mice with dietary obesity can eliminate the cognition and restore the immunoquiescence of microglia in hippocampus,^46^ proving the pivotal role of neuroinflammation. In the current study, our results showed that the expression levels of IL‐1β and IL‐6 were significantly increased in the POCD hippocampus, consistent with the previous reports. We also showed that EA treatment could decrease the expression of IL‐1β and IL‐6 in hippocampal tissues of POCD mice, which may serve as the mechanisms for EA‐afforded protection.

The activation and secretion of IL‐1β require Caspase‐1, whose activity is controlled by inflammasomes.^47^ The NLRP3 inflammasome was identified to play an important role in the mediated neuroinflammation.^48^ Growing studies have shown that NLRP3 inflammasomes were activated in POCD animals. Wang et al. reported that the activation of NLRP3 in aged mouse brain contributed to isoflurane‐induced hippocampal inflammation and cognitive impairment.^18^ Shao et al. showed that ChIV has a protective effects against sevoflurane‐induced neuroinflammation and cognitive dysfunction by inhibiting the NLRP3/caspase‐1 inflammasome pathway.^49^ Jin et al. reported that Baicalin improves cognitive impairment and protects neurons from microglia‐mediated neuroinflammation by inhibiting NLRP3 inflammasomes and TLR4/NF‐κB signaling pathway.^17^ These studies suggested that NLRP3‐mediated neuroinflammation plays an important role in cognitive impairment. In addition to NLRP3,^15^ ASC‐dependent mechanisms mediated by the NLRP1, AIM2, or NLRC4 inflammasomes were also confirmed to be involved in the neuroinflammation.^50^ Consistent with the previous reports, we also confirmed that the expressions of NLRP3 and AIM2 were increased in the POCD mice, while EA treatment reduced the expression of NLRP3, but not that of AIM2. It is well‐known that the activation of the NLRP3 inflammasome is regulated by the NF‐κB pathway.^15^ We also confirmed that EA treatment significantly decreased the phosphorylation of IκB‐α and p65 in the hippocampus, suggesting that EA improved POCD may through regulating NLRP3‐mediated neuroinflammation.

Previous study suggested that EA treatment could block the activation of NLRP3 inflammasome and the impairment of cognition in senescence‐accelerated mice.^51^ To further evaluate the necessity of NLRP3 in EA’s treatment effect, NLRP3 activator nigericin was applied to the POCD mice. Our data proved the effect of nigericin on NLRP3 as evidenced by increased NLRP3, ASC, and Caspase‐1. Interestingly, nigericin did not further enhance NLRP3 activation or aggravate cognitive dysfunction, suggesting that POCD itself is strong enough to induce NLRP3 activation and neuroinflammation. As expected, nigericin abolished the treatment effect of EA, confirming that EA works through NLRP3 inhibition.

Conclusively, we have proved in the present study that EA alleviates cognitive dysfunction associated with ameliorated neuroinflammation in aged POCD mouse model. Mechanistically, EA’s treatment effects are dependent on NLRP3 inhibition. EA may be a potentially translatable approach in managing POCD in aged population.

## CONFLICT OF INTEREST

The authors declare that they have no conflict of interest.

## AUTHOR CONTRIBUTIONS

Jiangang Song and Xing Li contributed to conception, supervision, and design of this article. Long Sun and Yue Yong involved in data analysis and editing the manuscript. Pan Wei, Yongqiang Wang, He Li, and Yalan Zhou involved in data collection. Wenqing Ruan contributed to establishment of predictive model. All authors contributed to the article and approved the submitted version.

## Supporting information

Fig S1

## Data Availability

The data that support the findings of this study are available from the corresponding author upon reasonable request.
