# Supplementary figures and images for: Electroacupuncture ameliorates postoperative cognitive dysfunction and associated neuroinflammation via NLRP3 signal inhibition in aged mice
Source: CNS Neurosci Ther. 2021 Dec 23;28(3):390–400. doi: 10.1111/cns.13784 (PMC8841296; doi:10.1111/cns.13784)

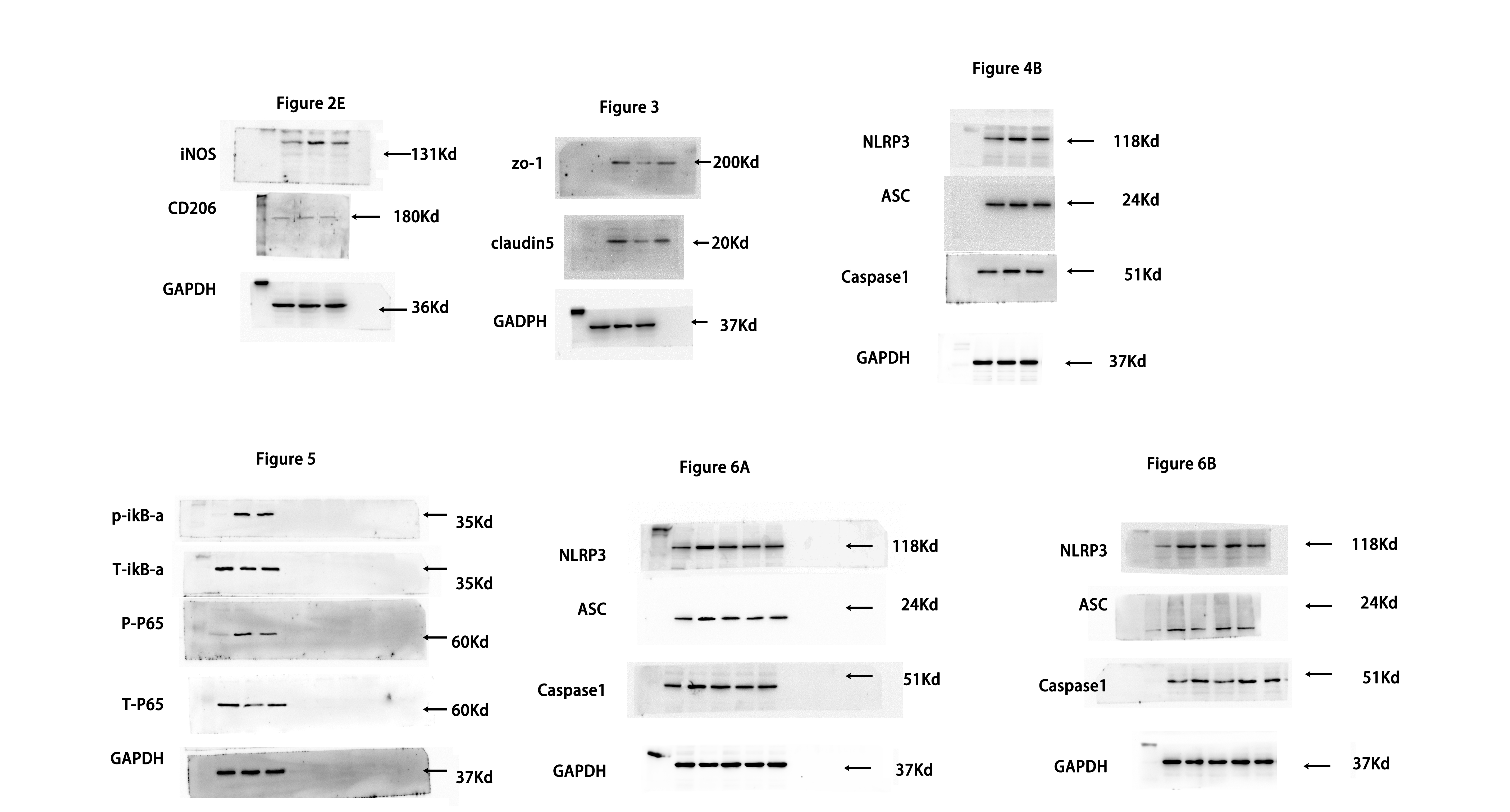

Supplement: Supplementary file 1 — Fig S1 [file CNS-28-390-s001.jpg]
